# Spontaneously Self-Assembled Microgel Film as Co-Delivery System for Skincare Applications

**DOI:** 10.3390/pharmaceutics13091422

**Published:** 2021-09-08

**Authors:** Garbine Aguirre, Pablo Taboada, Laurent Billon

**Affiliations:** 1 Institut des Sciences Analytiques & de PhysicoChimie pour l’Environnement & les Matériaux, Universite de Pau et des Pays de l’Adour, E2S UPPA, CNRS, UMR5254, 64000 Pau, France; laurent.billon@univ-pau.fr; 2 Bio-Inspired Materials Group, Functionalities & Self-Assembly, Universite de Pau et des Pays de l’Adour, E2S UPPA, Hélioparc, 2 Avenue Angot, 64000 Pau, France; 3 Particle Physics Department, Faculty of Physics, 15782 Campus Sur, University of Santiago de Compostela, 15782 Santiago de Compostela, Spain; pablo.taboada@usc.es

**Keywords:** self-assembled microgel film, cosmetic active molecules, co-delivery

## Abstract

Nowadays, the design of innovative delivery systems is driving new product developments in the field of skincare. In this regard, serving as potential candidates for on-demand drug delivery and fulfilling advanced mechanical and optical properties together with surface protection, spontaneously self-assembled microgel films can be proposed as ideal smart skincare systems. Currently, the high encapsulation of more than one drug simultaneously in a film is a very challenging task. Herein, different ratios (1:1, 3:1, 9:1) of different mixtures of hydrophilic/hydrophobic UVA/UVB-absorbers working together in synergy and used for skin protection were encapsulated efficiently into spontaneously self-assembled microgel films. In addition, in vitro release profiles show a controlled release of the different active molecules regulated by the pH and temperature of the medium. The analysis of the release mechanisms by the Peppas–Sahlin model indicated a superposition of diffusion-controlled and swelling-controlled releases. Finally, the distribution of active molecule mixtures into the film was studied by confocal Raman microscopy imaging corroborating the release profiles obtained.

## 1. Introduction

The efforts of the beauty and personal care industries to meet youthful, vibrant, and healthy skin criteria can be addressed through the smart delivery of skincare products. In this sense, even if the performance of the most effective products is boosted by the discovery of new active molecules, the design of new-targeted delivery systems is the backbone of the current research. Over the last few years, skincare applications, which require several simultaneous treatments, have directed their attention to advanced materials able to interact with the skin as smart delivery systems at the same time that they provided different advanced properties such as surface protection, mechanical and optical properties [1,2,3]. In the case of patients suffering from extreme skin injuries, recent advances are focused on the use of tissue engineering for the regeneration of damaged tissues. In this regard, particular attention is paid to the use of different biopolymers such as zein, chitosan, collagen, hyaluronic acid, gelatin and elastin, among others as a material matrix for scaffolds design [4,5,6].

Even if macroscopic gels have demonstrated interesting properties for skin, from the point of view of in-situ applications, their use is limited due to the inability to be integrated into healthcare formulations. This limitation can be overcome by the fascinating properties of microgels, definitively. Indeed, microgels are environmentally responsive cross-linked soft particles able to swell faster than macroscopic gels in a thermodynamically good solvent, responding to external stimuli such as temperature and pH, among others. Furthermore, due to the increase in the electrical double layer’s surface, they present a greatly larger surface area than that of macroscopic gels. Moreover, microgels are able to contain therapeutic molecules due to their sponge’s structure and release them in solution by changing their volumes [7], such as hydrophobic [8] and hydrophilic molecules [9], or even macromolecules [10], as shown for antitumor drug release systems [11,12].

From the point of view of skincare applications, the spontaneous formation of self-assembled microgel films without any external trigger is very useful. In this regard, many efforts have been made to form stable self-assembled microgel films through the creation of electrostatic or covalent interactions between the particles [13,14]. However, the protocols and microgels used based on poly(N-isopropylacrylamide) (PNIPAM), which is potentially toxic [15], are not suitable for skincare applications.

In this scenario, our group recently published the first reported oligo (ethylene glycol) (OEG) microgel [16] designed to spontaneously form a self-assembled and self-supported transparent film presenting similar mechanical deformations of the skin [17] via a simple solvent evaporation process at skin temperature and ambient pressure without any external triggers [18]. Moreover, interferential photonic and magnetic properties were provided to (OEG)-based microgels through the encapsulation of magnetic nanoparticles (NPs). In parallel, the encapsulation of different UV-absorbing molecules was studied [19]. The unusually high encapsulation of different types of cosmetically active molecules was achieved. In addition, loaded films presented UVA-UVB absorption as being useful for skin protection against the sun [20].

Currently, the research is focused on the use of delivery systems for combined encapsulation and delivery, i.e., more than one drug simultaneously encapsulated/released in the same carrier [21,22]. It has been reported that the reasonable combination of multiple drugs can significantly improve their expected effect reducing the dose needed for treatment and minimizing adverse reactions [23]. Therefore, in this work, we aim to evaluate the ability of (OEG)-based microgel self-assembled films to encapsulate different combinations of hydrophobic and hydrophilic cosmetic active molecules. Furthermore, the in vitro release profiles of the different cosmetic active molecules’ mixtures in response to pH, temperature and mixture type were analyzed. For this, release profiles were fitted to Peppas–Sahlin models and the distribution of different cosmetic active molecules was analyzed by confocal Raman spectroscopy imaging.

## 2. Materials and Methods

### 2.1. Materials

Di(ethylene glycol) methyl ether methacrylate (MeO2MA 95%, Aldrich), oligo(ethylene glycol) methyl ether methacrylate (OEGMA, terminated by 8 EG units with Mn = 475 g·mol^−1^), methacrylic acid (MAA) and oligo(ethylene glycol) diacrylate (OEGDA, number average weight Mn = 250 g·mol^−1^) were supplied by Sigma-Aldrich (St. Louis, MI, USA) and used without any purification. Potassium persulfate (KPS 99%, ABCR), as initiator, was used as received. Buffers were prepared using citric acid and sodium phosphate dibasic (Na_2_HPO_4_), both supplied by Sigma-Aldrich. Diethylamino hydroxybenzoyl hexyl benzoate (DBBH), benzophenone-4, and salicylic acid were kindly supplied by LVMH (St Jean de Braye, France). Purified water from the Millipore Milli-Q system (Lormont, France) and ethanol (VWR Chemicals, Rosny-sous-Bois, France) were used throughout the work.

### 2.2. Self-Assembled Microgel Films Formation

Prior to the film formation, oligo(ethylene glycol)-based microgels were synthesized following the method described by Boularas [24]. Then, self-assembled microgel films were formed following the procedure described before [19]. Briefly, self-assembled microgel films were formed via an easy handling procedure based on water evaporation under ambient conditions. An amount of 30 mL of non-purified (presence of water-soluble polymers, WSP) aqueous microgel dispersion (1.4 wt% solid content) was introduced into inert plastic molds and dried for 48 h at 35 °C (±3 °C) at atmospheric pressure.

### 2.3. In Vitro Encapsulation of Active Molecules Mixtures

The loaded amounts of different combinations of active molecules into films were determined by immersing the films in active molecule water/ethanol solution and allowing them to rehydrate for 24 h [19]. For the active molecule solutions preparations, different ratios (1:1, 3:1, 9:1) of hydrophilic/hydrophobic molecules were dissolved (total concentrations of the combinations were 500 µg/mg_film_ or 1 mg/mg_film_) in water/ethanol (50% of ethanol) solutions using magnetic stirring. For that and taking into account the active molecule amounts used throughout the work, DBBH was considered as a hydrophobic molecule and benzophenone-4 and salicylic acid as hydrophilic ones. Then, films were removed and the non-encapsulated active molecules in the supernatant were determined by UV-Vis. The experiments were performed in triplicate.

Entrapment efficiency (E.E.) was calculated as follows:

(1) $$E.E.\%=\frac{\mathrm{weight}\;\mathrm{of}\;\mathrm{active}\;\mathrm{molecule}\;\mathrm{in}\;\mathrm{microgel}\;\mathrm{dispersion}}{\mathrm{weight}\;\mathrm{of}\;\mathrm{feeding}\;\mathrm{active}\;\mathrm{molecule}}\times 100$$

### 2.4. Characterization of Loaded Films

Transmittance data of loaded films were collected using a Shimadzu UV-2101 spectrometer from 300 to 500 nm. Loaded films were sticky enough to adhere themselves to the sample holder and, therefore, air was used as reference.

Raman spectroscopy scans and images of the different types of self-assembled microgel films (bare and loaded ones) were obtained with a WITec Confocal Raman microscope model Alpha 300R + y (Ulm, Germany). Dried film samples were slightly wet with triple distilled water to homogenize them and avoid potential background fluorescence. In a typical experiment, surface and in-depth distribution of the different cosmetic active molecules along the films were determined using a frequency-doubled laser at 532 nm of excitation wavelength at an output power of 7 mW and 600 mm grating. Raman spectra for image compositions were recorded using a 100X Zeiss, EC Epiplan-Neofluar DIC objective (Oberkochen, Germany) with a numeric aperture of 0.9. Image resolution was set at 1024 × 127 pixels, with a total of 22,500 spectra per image at a scan speed of 20 s per line and an integration time per pixel of 0.13 s. Characteristic bands at 1203 and 479 cm^−1^, 1349 and 1080 cm^−1^, and 3072 and 774 cm^−1^ for benzophenone-4, DBBH and salicylic acid were used for identification, respectively (see Figure S1). Data acquisition was driven by the WITec Control software (Ulm, Germany). For peak identification in mixed films (cargo-loaded ones) Raman spectra of controls, that is, bare polymeric films and free cosmetic active molecules were recorded and compared.

### 2.5. In Vitro Active Molecules Mixtures Release from Self-Assembled Microgel Films

The effect of different parameters such as pH and temperature on the in vitro release of active molecules from self-assembled microgel films was evaluated. For that, loaded films with a 1 mg/mg_film_ of active molecule concentration were placed inside vials with 25 mL of different buffered media (pH 4.4 or 6) and incubated at different temperatures (25 and 37 °C). Samples of 2 mL were taken at different time intervals and the volume extracted was replaced with fresh buffer. The active molecule concentration in the release medium was determined by UV-Vis. All the experiments were performed in triplicate.

In addition, different kinetic models were studied to describe the experimental mixture release profiles from self-assembled microgel films. Firstly, the Korsmeyer–Peppas model [25] was investigated using the equation:

(2) MtM∞= ktn

secondly, Peppas–Sahlin model [26] was considered following the equation:

(3) MtM∞= k1tn+ k2t2n

where M_t_ and M_∞_ represent the amount of the active molecules released at time t and that initially contained in the formation, respectively, and k, k_1_ and k_2_ are the release rate coefficients.

## 3. Results and Discussion

Recently, we reported the kinetics of surfactant-free precipitation copolymerization of MeO_2_MA, OEGMA, MAA and OEGDA [27]. A homogeneous distribution of well-isolated carboxylic acid units inside microgel particles was observed. Moreover, microgel microstructure was analyzed by proton transverse relaxation (T_2_) measurements [16]. It was observed that microgel particles exhibited a core–shell structure, the cross-linking density of the core being five times higher than that of the shell. In addition, microgel particles presented low volume of the core (66%), this being one of the reasons of the spontaneous film formation properties presented by microgel particles.

Regarding the colloidal properties of the microgel, the study of the stimuli-responsiveness of the microgel synthesized was carried out at different temperatures and pH by dynamic light scattering [24]. For that, different buffered media at an ionic strength of 1 mM were used. The microgel showed the convectional thermal behavior at pH 6: upon heating above the volume phase transition temperature (VPTT) hydrodynamic diameter decreased due to the increase in hydrophobic interactions between nonpolar groups (see Figure S2a).

Regarding the pH-responsiveness of the microgel synthesized, this was analyzed at different values of pH and at swollen state (25 °C). The pH-sensitiveness was the expected one: below the volume phase transition pH (VPTpH) microgel particles were collapsed due to the protonation of carboxylic groups and the absence of electrostatic repulsion and above it, particles were swollen due to the charge repulsion in the polymer network caused by the ionization of the carboxylic groups. (see Figure S2b).

In this study, self-assembled microgel films were formed by a simple solvent evaporation process [19]. It was observed that the self-assembled microgel films presented a reversible swelling/de-swelling behavior without losing their identity due to the cohesive properties among microgel particles. In addition, their swelling ability could be controlled through the hydrophobicity of the medium as well as the temperature (see Figure S3).

### 3.1. Active Molecules Mixtures Encapsulation into Self-Assembled Microgel Films

The use of thin films as delivery systems could be largely limited due to their low drug encapsulation capacity [28,29]. In addition, the combination of more than one drug simultaneously is a very challenging task due to the difficulty to obtain perfectly reproducible loaded films from batch to batch in terms of drug incorporation/encapsulation, which may lead to different biological outcomes and even to toxic effects [30,31]. In the present work, different active molecules mixtures loaded into self-assembled microgel films were studied taking the advantage of the swelling ability of the films in water/ethanol mixtures (Figure S3) [19]. Self-assembled microgel films were rehydrated in water/ethanol (50%) mixtures containing different concentrations of different active molecules mixtures for 24 h. Mixture compositions were based on the hydrophobicity/hydrophilicity of the active molecules of DBBH, i.e., the hydrophobic molecule, and benzophenone-4 and salicylic acid, i.e., the hydrophilic ones. In Table 1, the average encapsulation efficiencies (E.E.) of different mixtures (hydrophilic/hydrophobic ratio was 1:1) are shown. As can be seen, in the case of mixtures formed with hydrophobic and hydrophilic molecules, the E.E. values were rather low (E.E. < 60%), the encapsulation of the hydrophobic one (DBBH) being higher in all cases. In addition, the decrease in transmittance was only observed at the characteristic wavelength of the hydrophobic molecule (DBBH) but not at that of hydrophilic ones (benzophenone-4 and salicylic acid) (see Figure S3). It seems that the hydrophobicity of the DBBH molecule has an effect on the effective encapsulation of hydrophilic ones (benzophenone-4 and salicylic acid). By contrast, the results obtained in the case of the mixtures formed with two hydrophilic molecules, benzophenone-4 and salicylic acid, showed an effective E.E. of both active molecules and synergy effect in terms of the decrease in the transmittance between the characteristics wavelengths of both molecules. Previously, our group described, using NOESY-NMR measurements, that hydrophobic interactions between ethylene/methylene groups of the films and aromatic rings of active molecules as well as H-bonding interactions between the –OH groups of active molecules and the ether oxygen of the ethylene glycol units of the self-assembled microgel films were responsible for the high encapsulation of the molecules studied [19]. In the same way, it was reported that the release of hydrophilic active molecules (benzophenone-4 and salicylic acid) was enhanced above VPTT (hydrophobic state of the microgels) due to the decrease in the short-distance hydrophobic interactions between hydrophilic active molecules and microgel particles [27]. Therefore, after DBBH encapsulation the enhanced hydrophobicity of the self-assembled microgel film can hinder the efficient encapsulation of both hydrophilic active molecules.

In addition, as can be seen in Table 1, E.E. values increased for all the mixtures studied as the concentration of the active molecule increased, with the encapsulation of DBBH (hydrophobic one) higher in all cases. The results obtained suggested that above mentioned hydrophobic and H-bonding interactions between self-assembled microgel films and active molecules induced intermolecular complexes formation obtaining high E.E. values [32].

With the aim of improving the E.E. values of hydrophilic active molecules and to confirm the above hypothesis, different ratios of hydrophilic/hydrophobic molecules (3:1 and 9:1) were studied for mixtures A and B. The new mixtures with the ratio 3:1 were renamed as mixture A-3 and mixture B-3 and the ones with the ratio 9:1 as mixture A-9 and mixture B-9. The total amount of active molecules was maintained at 1 mg/mg_film_, in all cases. Decreasing the concentration of hydrophobic molecules, the E.E. values increased for hydrophilic but also for hydrophobic molecules, in the case of both mixtures (Table 2). The total amount of active molecules loaded was higher than that observed by Boateng et al. for paracetamol and amoxicillin using carboxymethyl cellulose, carrageenan and sodium alginate-based films [29]. In addition, the results obtained suggested that the hydrophobicity of the DBBH hindered the encapsulation of hydrophilic active molecules into the film. Xu and coworkers reported the simultaneous encapsulation of hydrophilic and hydrophobic drugs into poly(N-isopropyl acrylamide)-b-poly(stearyl methacrylate) (PNIPAM-PSMA) nanoparticles [33]. They observed that the encapsulation of both drugs was mostly dependent on respective interactions between polymer and drugs. In addition, it was reported that increasing the hydrophobicity of the polymeric nanoparticles hindered hydrogen bond formation between the hydrophilic drug and nanoparticles decreasing its loading amount. Then, the increase in the hydrophobicity of the film because of the huge amount of DBBH molecules loaded could render hydrogen bonding difficult between hydrophilic active molecules and film hindering their efficient encapsulation. It is interesting to point out that loaded films are able to stop UVA and UVB radiations (see Figure S4), being able to stop the global UV radiations and act as skin protectors against the sun.

### 3.2. In Vitro Release of Mixtures from Self-Assembled Microgel Films

**Figure 1:**
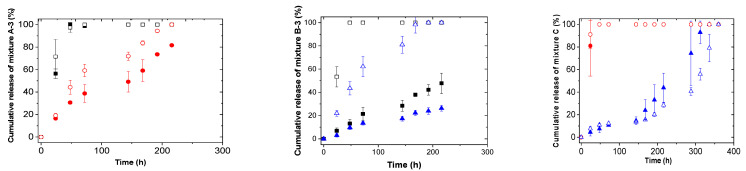
Release kinetics as a function of pH for different mixtures at 25 °C. ■ DBBH at pH 6, □ DBBH at pH 4.4, ● Benzophenone-4 at pH 6, ○ Benzophenone at pH 4.4, ▲ Salicylic acid at pH 6, Δ Salicylic acid at pH 4.4.

**Figure 2:**
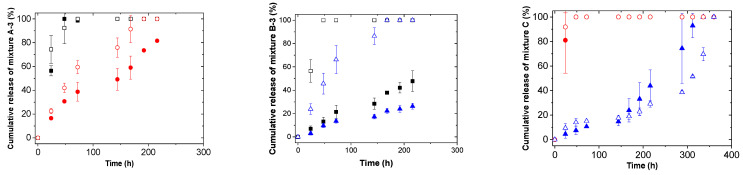
Release kinetics as a function of temperature for different mixtures at pH 6. ■ DBBH at 25 °C, □ DBBH at 37 °C, ● Benzophenone-4 at 25 °C, ○ Benzophenone at 37 °C, ▲ Salicylic acid at 25 °C, Δ Salicylic acid at 37 °C.

The study of different mixtures release kinetics was carried out with varying different parameters such as the temperature and pH of the medium. For that, the same concentration of mixtures (1 mg/mg_film_) was loaded but increasing the ratio of the hydrophilic molecule to 3:1 in the case of mixtures A and B, mixture A-3 and mixture B-3, respectively. In this way, the E.E. values were above 70% for all active molecules. Previously, our group observed that both types of molecules, i.e., hydrophobic and hydrophilic, were forming different types of aggregates inside self-assembled microgel films [19]. Prior to the diffusion of active molecules from films to the release medium, the dissolution of them should happen. For that and with the aim of enhancing the dissolution of the aggregates and their subsequent release, a water/ethanol (50:50) release medium was used. It is important to point out that all active molecules used in the present work are soluble in ethanol, the last one being widely used in medicinal or cosmetic products as well as pharmaceutical preparations for direct human skin applications [34]. As can be seen in Figure 1 and Figure 2, the complete release of active molecules was obtained. In addition, changing the release medium parameters, i.e., pH and temperature, and the combination of active molecules made, allowed the control of the release kinetics. In this sense, the release of the different active molecules was higher under those conditions where the hydrophobicity of the films was enhanced (37 °C or pH 4.4). Our group reported that the swelling ability of self-assembled microgel films in hydrophobic media was higher above the VPTT when films are hydrophobic. Therefore, the observed enhanced release of different active molecules at pH 4.4 or 37 °C could be related to the higher swelling of the films together with the higher solubility of active molecules in water/ethanol medium. It is important to remark that the release profiles obtained for active molecules at those conditions in which the films were hydrophilic (pH 6 and 25 °C) and/or hydrophobic (pH 4.4 and 37 °C) were rather similar. These results confirmed the reproducibility of release kinetics of active molecules from both types of films. In addition, the release of benzophenone-4 was enhanced when combined with salicylic acid instead of DBBH. Moreover, an inhibition time of 200 h was observed for salicylic acid in the case of mixture C with a 1/1 ratio of benzophenone-4 and salicylic acid.

**Figure 3:**
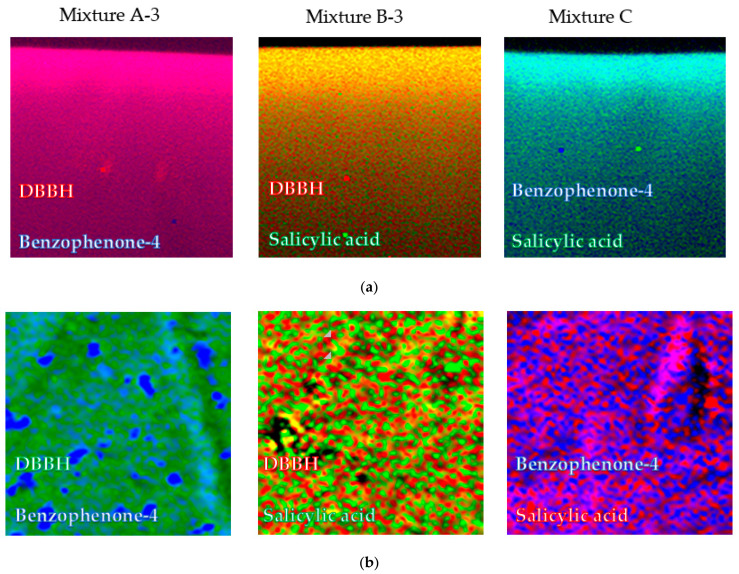
Cross-sectional from the top (image top) to the bulk (image bottom) (50 × 50 µm^2^) (**a**) and 2D surface (75 × 75 µm^2^) (**b**) confocal images of different mixtures.

It is reported that the release profiles are dependent on the distribution/location of active molecules into films and/or microgels [35]. In order to corroborate the release kinetics obtained and to analyze the internal distribution of different UV-absorbing molecules, confocal Raman spectroscopy imaging experiments were performed on the films loaded with different active molecule mixtures. For that, dual-drug loaded films with the same concentration of mixtures (1 mg/mg_film_) and same hydrophilic/hydrophobic molecules ratios (3:1 in the case of mixtures A and B and 1:1 for mixture C) used during in vitro release experiments were analyzed (mixture A-3, mixture B-3 and mixture C). Figure 3 shows the distribution of each active molecule for different mixtures along the *z*-axis of the film, i.e., thickness (from top to bottom of the self-assembled film, Figure 3a) and in the surface (Figure 3b). A characteristic color for each active molecule was used for the different types of dual-drug loaded films. It is important to remark that, after repeating the measurements in different areas of the samples and using different specific bands of the active molecules, the same results were obtained (data not shown). As can be observed in Figure 3, the images seem to be in accordance with the behavior observed in the in vitro release profiles. In this sense, as can be seen in Figure 3, in the case of mixtures A-3 and B-3, the most hydrophobic molecule (DBBH) of the mixtures was located at the surface, preferentially, their release being faster, even through a burst phase. This was particularly observed for the heterogeneous surface of mixture A-3 loaded film (Figure 3b) where islands of benzophenone-4 (hydrophilic) were observed on a surface fully covered with DBBH (hydrophobic). By contrast, in the case of mixture B-3 (DBBH and salicylic acid), a more homogeneous distribution of both molecules at the film surface was observed (Figure 3) in spite of the faster release observed for the hydrophobic DBBH molecule. Finally, in the case of mixture C formed with both hydrophilic active molecules (benzophenone-4 and salicylic acid), the presence of benzophenone-4 was predominant at the film surface, again in agreement with its enhanced release as observed above. The same behavior was observed by Boateng et al. for films loaded with paracetamol and amoxicillin [29]. Even both drugs were hydrophilic, a faster release rate was observed for paracetamol than for amoxicillin due to its higher presence on the surface of the films.

As mentioned previously, it seems that the distribution of the active molecules into the film was dependent on their hydrophilic/hydrophobic character. In this regard, a more homogeneous distribution of hydrophilic molecules was observed throughout the film than that of hydrophobic ones [19]. This could be related to the presence of water molecules in the “dry” films, as explained in our previous work [19]. The drying process together with the presence of water molecules into the film could induce a displacement of hydrophobic molecules to the surface of the films enhancing their release.

With the aim of better understanding the release mechanisms of different mixtures, the release profiles were fitted to Korsmeyer–Peppas [25] and Peppas–Sahlin models [26]. It is important to note that the extreme values of release exponent (*n*), which is indicative of the mechanism of drug release, are a function of the geometry of the delivery system. In this sense, Siepmann et al. reported that for thin films those extreme values are 0.5 and 1 [36]. Briefly, *n* = 0.5 indicates diffusion-controlled drug release and *n* = 1 indicates swelling-controlled drug release. Values of *n* between 0.5 and 1 are related to the superposition of both phenomena [36]. From Korsmeyer–Peppas model *n* < 0.5 values were obtained for the most hydrophobic components (DBBH and benzophenone-4) into the mixtures, suggesting the diffusion-controlled release of them (Tables S1 and S2). However, even if the values of the exponent n obtained would indicate a diffusion-controlled drug release mechanism, this was not valid due to the low R^2^ values obtained. In the case of more hydrophilic components into the mixtures, values of *n* between 0.5 and 1 were obtained, except for salicylic acid mixture C at 25 °C/pH 6, suggesting the superposition of diffusion and swelling controlled mechanisms. Similar release exponents were reported by Boateng et al. for hydrophilic paracetamol release from sodium carboxymethylcellulose wafers and films [37]. Even if they observed faster paracetamol release from wafers than from corresponding films, release exponent values between 0.5 and 1.2 were obtained for all the formulations suggesting a combined release process between diffusion and hydration of the wafers/films.

With the aim of increasing the accuracy of the fitting model and obtaining more information about the mechanism of mixtures release, Peppas–Sahlin model was used to fit the release profiles. Using Peppas–Sahlin model it is possible to calculate the contribution of diffusion-controlled and swelling-controlled mechanisms [26]. After applying Peppas–Sahlin model, a superposition of both mechanisms was observed in all cases except for salicylic acid (mixture C) at 25 °C/pH4.4 and 37 °C/pH6 conditions (Tables S3 and S4). At those conditions, a value of *n* > 1 was obtained, surprisingly. It is important to point out that for this combination of active molecules and for the fitting of salicylic acid release profiles, the first inhibition period was avoided. Perhaps this could be the reason for those unexpected release exponent values. In addition, *k*1 values were higher than *k*2 ones suggesting Fickian diffusion as the predominant release mechanism. This was an expected result taking into account the non-covalent nature of interactions between active molecules and self-assembled microgel films. The same behavior was reported by Silva et al. for chitosan-based nano emulsion-loaded films for transdermal delivery [38]. In this case, the release of methyl salicylate drug was governed by Case II transport, i.e., drug diffusion together with swelling of the polymeric chitosan network.

## 4. Conclusions

In summary, the usefulness of self-assembled oligo(ethylene glycol)-based microgel films as co-delivery systems was demonstrated. Films were able to encapsulate different combinations of hydrophobic/hydrophilic active molecules at high amounts (1 mg/mg_film_). After studying the in vitro release of different mixtures of active molecules from the film, it was observed that the release of each active molecule could be triggered by the pH, temperature and combination made. In addition, the existence of a superposition of Fickian diffusion and Case-II transport mechanisms was observed with the Fickian diffusion as the predominant one. From the point of view of future film applicability, thanks to the high co-loading of different active molecules combinations together with their controlled co-delivery and film-formation on skin, this platform offers a sophisticated delivery tool for healthcare and medical applications. The next step in this regard will be the application of proposed films to ex vivo skin models with the aim of studying their toxicity and ability of controlled co-delivery at skin conditions (temperature and pH).

## Figures and Tables

**Table 1:** Encapsulation efficiency (E.E.) values for different mixtures.

Loaded Amount (mg/mg_film_)	Encapsulation Efficiencies (E.E.) %					
	Mixture A		Mixture B		Mixture C	
	DBBH	Benzophenone-4	DBBH	Salicylic Acid	Benzophenone-4	Salicylic Acid
0.5	23.0 ± 11.3	0	26.4 ± 9.1	2.8 ± 3.9	28.6 ± 1.3	51.4 ± 0.8
1	57.0 ± 2.0	19.0 ± 3.0	57.0 ± 5.3	21.50 ± 5.4	73.2 ± 0.4	87.4 ± 0.5

**Table 2:** Encapsulation efficiency (E.E.) values for different mixtures and different hydrophilic/hydrophobic ratios.

Hydrophilic/ Hydrophobic Ratio	Encapsulation Efficiencies (E.E.) %			
	Mixture A		Mixture B	
	DBBH	Benzophenone-4	DBBH	Salicylic Acid
3:1	73.0 ± 1.7	81.1 ± 0.4	80.5 ± 0.4	87.2 ± 0.2
9:1	57.3 ± 1.7	87.30 ± 0.6	83.10 ± 1.9	90.3 ± 0.8

## Data Availability

Not applicable.

## Supplementary Materials

The following are available online at https://www.mdpi.com/article/10.3390/pharmaceutics13091422/s1, Figure S1. Raman spectra of loaded films: bare film (black line), DBBH (red line), benzophenone-4 (blue line), and salicylic acid (green line Figure S2: Average hydrodynamic diameters as a function of temperature (a) and pH (b) in buffered media with an ionic strength of 1mM. Figure S3: Swelling ratio of self-assembled microgel film at 20 (■) and 50 °C (□) as a function of ethanol %. Figure S4: Optical transmission as a function of wavelength of films loaded with mixture 1 (DBBH and benzophenone-4) (a) and mixture 2 (DBBH and salicylic acid) (b). Table S1: Fitting parameters for Korsmeyer–Peppas model as a function of pH for mixture 1 (a), mixture 2 (b), and mixture 3 (c) mixtures.). Table S2: Fitting parameters for Korsmeyer–Peppas model as a function of temperature for mixture 1 (a), mixture 2 (b), and mixture 3 (c) mixtures. Table S3: Fitting parameters for Peppas–Sahlin model as a function of pH for mixture 1 (a), mixture 2 (b), and mixture 3 (c) mixtures.). Table S4: Fitting parameters for Peppas–Sahlin model as a function of temperature for mixture 1 (a), mixture 2 (b), and mixture 3 (c) mixtures.
