# Supplementary material for: Spontaneously Self-Assembled Microgel Film as Co-Delivery System for Skincare Applications
Source: Pharmaceutics. 2021 Sep 8;13(9):1422. doi: 10.3390/pharmaceutics13091422 (PMC8472779; doi:10.3390/pharmaceutics13091422)
Supplement: Supplementary file 1 [file pharmaceutics-13-01422-s001.zip › pharmaceutics-1374503-SM.pdf]

pharmaceutics

Supplementary Material: Spontaneously Self-Assembled Microgel Film as Co-Delivery System for Skincare Applications

Garbine Aguirre, Pablo Taboada and Laurent Billon

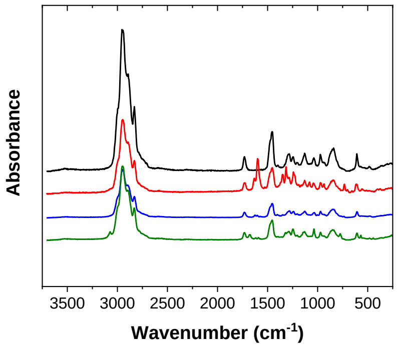

Figure S1. Raman spectra of single loaded-films: bare film (black line), DBBH (red line), benzophenone-4 (blue line), and salicylic acid (green line).

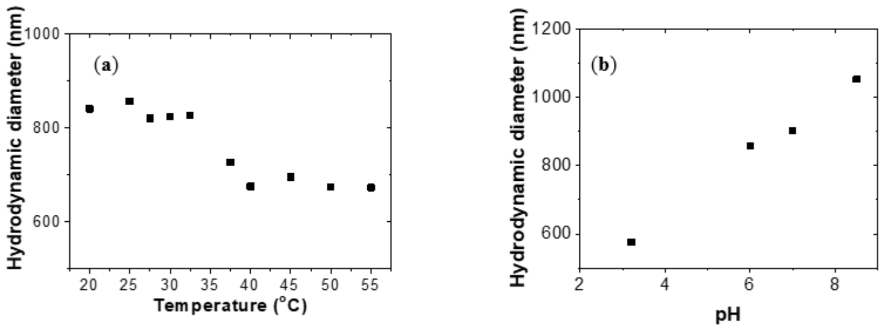

Figure S2. Average hydrodynamic diameters as a function of temperature (**a**) and pH (**b**) in buffered media with an ionic Scheme 1. mM.

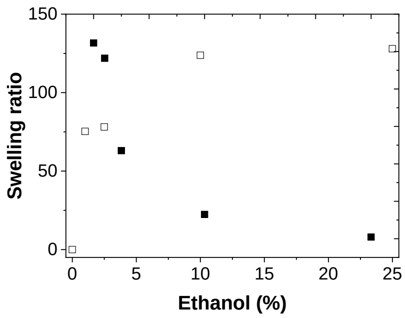

Figure S3. Swelling ratio of self-assembled microgel film at 20 (■) and 50 °C (□) as a function of ethanol %.

Citation: Aguirre, G.; Taboada, P.; Billon, L. Spontaneously Self-Assembled Microgel Film as Co-Delivery System for Skincare Applications. *Pharmaceutics* **2021**, *13*, 1442. https://doi.org/10.3390/pharmaceutics13091422

Academic Editor: Bozena B. Michniak-Kohn

Received: 25 August 2021
Accepted: 7 September 2021
Published: 8 September 2021

Publisher's Note: MDPI stays neutral with regard to jurisdictional claims in published maps and institutional affiliations.

Copyright: © 2021 by the authors. Submitted for possible open access publication under the terms and conditions of the Creative Commons Attribution (CC BY) license (http://creativecommons.org/licenses/by/4.0/).

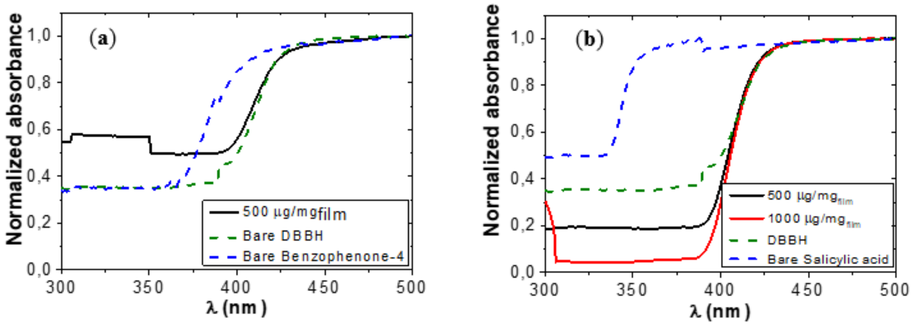

Figure S4. Optical transmission as a function of wavelength of films loaded with mixture (DBBH and benzophenone-4) (**a**) and mixture (DBBH and salicylic acid) (**b**).

Table S1. Fitting parameters for Korsmeyer-Peppas model as a function of pH for mixture A-3 (a), mixture B-3 (b), and mixture C (c) mixtures.

	Fitting parameters	DBBH		Benzophenone-4	
		pH 4.5	pH 6	pH 4.5	pH 6
**(a)**	k	1.74 ± 0.08	1.57 ± 0.15	0.43 ± 0.15	0.33 ± 0.11
	n	0.12 ±0.04	0.20 ± 0.08	0.68 ± 0.08	0.66 ± 0.05
	R^2	0.62	0.57	0.94	0.97

	Fitting parameters	DBBH		Salicylic acid	
		pH 4.5	pH 6	pH 4.5	pH 6
**(b)**	k	1.53 ± 0.17	−0.30 ± 0.10	0.47 ± 0.16	−0.63 ± 0.22
	n	0.21 ± 0.09	0.84 ± 0.05	0.68 ± 0.06	0.89 ± 0.11
	R^2	0.56	0.98	0.97	0.93

	Fitting parameters	Benzophenone-4		Salicylic acid	
		pH 4.5	pH 6	pH 4.5	pH 6
**(c)**	k	1.95 ± 0.02	1.88 ± 0.04	−0.47 ± 0.34	−1.18 ± 0.25
	n	0.02 ± 0.01	0.05 ± 0.02	0.86 ± 0.16	1.21 ± 0.11
	R^2	0.48	0.48	0.77	0.93

Table S2. Fitting parameters for Korsmeyer-Peppas model as a function of temperature for mixture A-3 (a),mixture B-3 (b), and mixture C (c) mixtures.

	Fitting parameters	DBBH		Benzophenone-4	
**(a)**		25 °C	37 °C	25 °C	37 °C

k	1.57 ± 0.15	1.75 ± 0.06	0.33 ± 0.11	0.49 ± 0.09
n	0.20 ± 0.08	0.11 ± 0.03	0.66 ± 0.05	0.66 ± 0.05
R^2	0.57	0.73	0.97	0.98

(b)

**Fitting parameters**	**DBBH**		**Salicylic acid**	
	**25 °C**	**37 °C**	**25 °C**	**37 °C**
k	−0.30 ± 0.10	1.57 ± 0.16	−0.63 ± 0.22	0.55 ± 0.12
n	0.84 ± 0.05	0.19 ± 0.08	0.89 ± 0.11	0.64 ± 0.06
R^2	0.98	0.56	0.93	0.96

(c)

**Fitting parameters**	**Benzophenone-4**		**Salicylic acid**	
	**25 °C**	**37 °C**	**25 °C**	**37 °C**
k	1.88 ± 0.04	1.95 ± 0.02	−1.18 ± 0.25	−0.16 ± 0.29
n	0.05 ± 0.02	0.02 ± 0.01	1.21 ± 0.11	0.73 ± 0.13
R^2	0.48	0.47	0.93	0.77

Table S3. Fitting parameters for Peppas-Sahlin model as a function of pH for mixture A-3 (a), mixture B-3 (b), and mixture C (c) mixtures.

(a)

**Fitting parameters**	**DBBH**		**Benzophenone-4**	
	**pH 4.5**	**pH 6**	**pH 4.5**	**pH 6**
k_1	0.86 ± 0.11	0.14 ± 0.07	0.03 ± 0.01	0.03 ± 0.01
k_2	−0.1875 ± 0.0519	−0.0048 ± 0.0015	−0.0015 ± 0.0013	0.0010 ± 0.0008
n	0.17 ±0.03	0.55 ± 0.11	0.69 ± 0.23	0.53 ± 0.10
R^2	0.99	0.95	0.95	0.93

(b)

**Fitting parameters**	**DBBH**		**Salicylic acid**	
	**pH 4.5**	**pH 6**	**pH 4.5**	**pH 6**
k_1	0.09 ± 0.04	0.08 ± 0.01	0.02 ± 0.01	0.03 ± 0.001

	k_2	−0.0019 ± 0.0010	−0.0013 ± 0.0009	−0.0001 ± 0.0001	−0.0001 ± 0.0001
	n	0.64 ± 0.12	0.67 ± 0.10	0.76 ± 0.15	0.74 ± 0.15
	R^2	0.95	0.96	0.99	0.99

	**Fitting parameters**	**Benzophenone-4**		**Salicylic acid**	
		**pH 4.5**	**pH 6**	**pH 4.5**	**pH 6**
**(c)**	k_1	0.86 ± 0.08	0.56 ± 0.09	2.74 e^{-6} ± 1.32 e^{-6}	−0.46 ± 0.09
	k_2	−0.1849 ± 0.0437	−0.0783 ± 0.0273	1.407 e^{-11} ± 0.925 e^{-11}	0.1112 ± 0.0317
	n	0.16 ± 0.02	0.25 ± 0.04	2.06 ± 0.10	0.30 ± 0.08
	R^2	0.99	0.99	0.96	0.98

Table S4. Fitting parameters for Peppas-Sahlin model as a function of temperature for mixture A-3 (a),mixture B-3 (b), and mixture C (c) mixtures.

	**Fitting parameters**	**DBBH**		**Benzophenone-4**	
		**25 °C**	**37 °C**	**25 °C**	**37 °C**
**(a)**	k_1	0.14 ± 0.07	0.47 ± 0.14	0.03 ± 0.01	0.02 ± 0.01
	k_2	−0.0048 ± 0.0015	−0.0541 ± 0.0032	0.0010 ± 0.0008	−0.0001 ± 0.0001
	n	0.55 ± 0.11	0.30 ± 0.06	0.53 ± 0.10	0.77 ± 0.16
	R^2	0.95	0.71	0.93	0.97

	**Fitting parameters**	**DBBH**		**Salicylic acid**	
		**25 °C**	**37 °C**	**25 °C**	**37 °C**
**(b)**	k_1	0.08 ± 0.01	0.18 ± 0.01	0.03 ± 0.001	0.02 ± 0.01
	k_2	−0.0013 ± 0.0009	−0.0079 ± 0.0066	−0.0001 ± 0.0001	−0.0001 ± 0.0001
	n	0.67 ± 0.10	0.51 ± 0.09	0.74 ± 0.15	0.92 ± 0.25
	R^2	0.96	0.96	0.99	0.99

	**Fitting parameters**	**Benzophenone-4**		**Salicylic acid**	
**(c)**		**25 °C**	**37 °C**	**25 °C**	**37 °C**

k_1	0.56 ± 0.09	0.90 ± 0.08	−0.46 ± 0.09	6.64 e^{-6} ± 2.5 e^{-6}
k_2	−0.0783 ± 0.0273	−0.1999 ± 0.0482	0.1112 ± 0.0317	8.177 e^{-11} ± 1.966 e^{-11}
n	0.25 ± 0.04	0.16 ± 0.02	0.30 ± 0.08	1.90 ± 1.60
R^2	0.99	0.99	0.98	0.89
